# Specifics of anticipatory competence of adolescents with motor disorders

**DOI:** 10.1192/j.eurpsy.2025.1900

**Published:** 2025-08-26

**Authors:** A. Akhmetzyanova, T. Artemyeva, V. Nikiforova

**Affiliations:** 1 Department of Psychology and Pedagogy of Special Education, Kazan Federal University, Kazan, Russian Federation

## Abstract

**Introduction:**

Anticipatory competence allows an adolescent to act and make important decisions in life and activities with a certain temporal-spatial foresight. However, due to their disorder, adolescents with motor impairments are not always able to anticipate the development of future events, and they experience difficulties in establishing social contacts and understanding the emotional states of others.

**Objectives:**

To study the specifics of anticipatory competence in adolescents with motor disorders.

**Methods:**

The study involved 46 adolescents (aged 12-15) attending educational institutions for children with disabilities, with a history of spastic diplegia, spastic tetraplegia, and ataxia. The research employed the following methods: the “Achenbach’s Questionnaire,” V.D. Mendelevich’s “Test of Anticipatory Competence,” V.P. Ulyanova’s “Anticipation of the Outcome of a Situation with Norm Violations,” and the authors’ method “Study of Anticipatory Competence of Adolescents” by A.I. Akhmetzyanova and T.V. Artemyeva.

**Results:**

The study revealed that, although the overall level of anticipatory competence in adolescents with motor disorders is quite high, these children experience difficulties in spatial orientation and in structuring a sequence of actions. They encounter challenges in planning future activities and they frequently experience delays. Adolescents with this pathology demonstrated low anticipatory competence in virtual reality, indicating difficulties in virtual communication with others.

**Conclusions:**

The findings of this study on the specifics of anticipatory competence in adolescents with motor disorders will help develop and implement programs for the social adaptation of children, preparing them for future professional activities and independent living in the community.

This paper has been supported by the Kazan Federal University Strategic Academic Leadership Program (PRIORITY-2030).

**Disclosure of Interest:**

None Declared

